# Policing the pandemic in Scotland: Using administrative data to measure underlying inequalities

**DOI:** 10.23889/ijpds.v8i2.2196

**Published:** 2023-09-14

**Authors:** Susan McVie, Victoria Gorton, Ben Matthews

**Affiliations:** 1 University of Edinburgh, Edinburgh, United Kingdom; 2 University of Stirling, Stirling, United Kingdom

## Objectives

In March 2020, police were given temporary powers to enforce the new Coronavirus Health Protection Regulations.

Our research asks:

1. How was non-compliance (as measured through police-issued fines) related to other underlying health and social inequalities?
2. What level of COVID-19 risk did those who failed to comply pose?

## Method

This project securely links, at an individual level, a dataset of all COVID fines issued by Police Scotland to a range of health and social administrative data. This data includes: information from healthcare settings on mental ill health, alcohol problems and drug addictions; geographic data on household type and occupancy; and government data on deprivation and deaths. The dataset has also been linked to a matched sample of controls (individuals who were not fined), and to COVID testing data, allowing us to examine the extent to which individuals who were fined posed a health risk to wider society.

## Results

Our results show that those who were fined were more likely to be living with underlying health and social inequalities than the matched controls. However, these differences did reduce over the course of the pandemic, most likely reflecting a combination of changing policing practice and weakening public compliance with the restrictions. Although not without limitations, testing data suggests that those who were fined were not substantially more likely to test positive for COVID during the pandemic than matched controls who were not fined. This raises questions about the role of police enforcement during a pandemic.

## Conclusion

This research is the first time police data has been linked to health and social care data in Scotland. Our findings demonstrate the considerable value of data linkage for understanding the challenges of policing public health whilst also allowing the empirical evaluation of policy decisions at scale.

